# Supplementary material for: A radial map of multi-whisker correlation selectivity in the rat barrel cortex
Source: Nat Commun. 2016 Nov 21;7:13528. doi: 10.1038/ncomms13528 (PMC5121329; doi:10.1038/ncomms13528)
Supplement: Supplementary Information — Supplementary Figures 1-7 [file ncomms13528-s1.pdf]

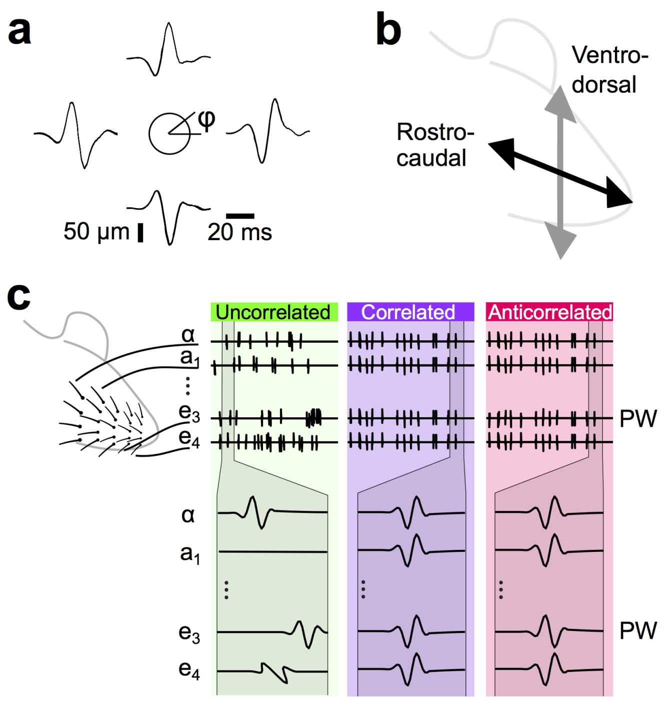

Supplementary Figure 1 Multi-whisker stimulus design. On each of the 24 largest whiskers on the right side of the rat (straddlers and columns 1 to 4, rows A to E), a series of discrete deflections were applied. Individual deflections were drawn in random order from a series of eight different shapes defined by the phase of the sinusoidal carrier oscillation stimulus and the direction of the deflection. (**a**) Average of 10 readings by a laser telemeter of the 4 different applied phases. (**b**) These stimuli were applied along two axes, either rostro-caudal or ventro-dorsal, leading to a total of 8 stimuli. (**c**) Each of the 24 largest whiskers of the rat received statistically identical stimulation sweeps, where individual stimuli occurred following a Poisson distribution (time constant: 500 ms, refractory period: 40 ms). However, at the multi-whisker level, different stimuli were generated. Top: time course of correlated, uncorrelated and anti-correlated stimuli applied to the rat whisker pad. Bottom: Time close-up on individual deflections across whiskers. Tilted deflections are those applied on the rostro-caudal axis. Straight ones correspond to those on the ventro-dorsal axis. By applying the same realization of the Poisson train on all whiskers, the resulting stimulus was correlated (purple, middle). By applying a different realization of the stimulus on each whisker, an uncorrelated stimulus was generated (green, left). Finally, by applying opposite directions of stimulation to the principal whisker versus all others, an anti-correlated stimulus was generated (pink, right).

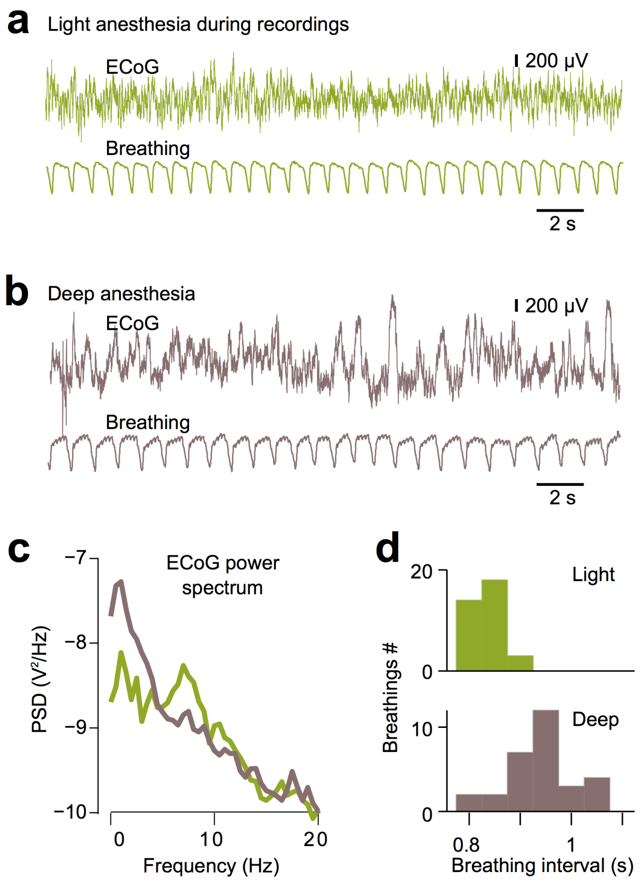

Supplementary Figure 2 Maintenance of light anesthesia during recordings. To ensure that a light anesthesia (stage III, plane 1–2) was maintained during the recordings of functional activity, an epidural ECoG electrode was implanted in a second craniotomy rostral to the main imaging craniotomy. In addition, to assess breathing frequency and regularity, a non-invasive piezo-electric breathing sensor was positioned under the rat belly. Both parameters were tracked over time. We maintained a desynchronized ECoG and a regular, 1-1.5 Hz breathing pattern, by adjusting the isoflurane concentration in the O_2/N_2O breathing mix. (**a**) Example of light anesthesia used during recordings, based on ECoG activity and breathing pattern. (**b**) Overly deep anesthesia. (**c**) ECoG power spectrum during light (green) and deep (brown) anesthesia. (**d**) Inter-breath interval distribution during light and deep anesthesia.

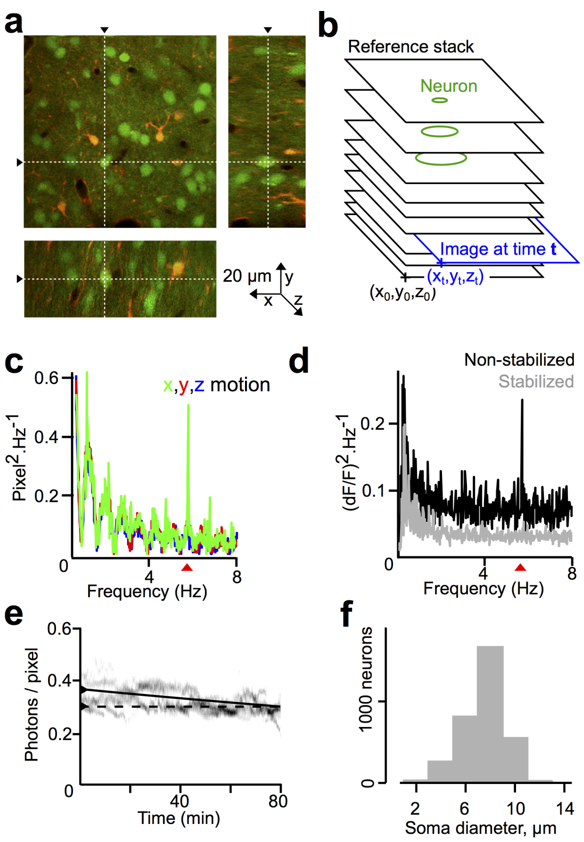

Supplementary Figure 3 Two-photon imaging of OGB-1 and SR101-stained cortex. (**a**) Representative example of a field of view acquired in layer 2/3 of the barrel cortex. Green channel: bolus loaded OGB1 fluorescent calcium indicator. Red channel: SR101 marked astrocytes. (**b**) Principle of the matching of ongoing images of OGB1 fluorescence (blue) with the reference stack (black). (**c**) On the same experiment as in (**a**), power spectral density of the movements of the acquired images during one recording, with respect to the reference stack. Notice high power at heart beat rate (red triangle). (**d**) On the same example, average power spectral density of calcium signals extracted from identified neuron ROI, without (black) and with movement registration (grey). Notice the disappearance of the heart beat component (red triangle) and the general decrease of power due to the removal of fluorescence fluctuations related to movement. (**e**) Raw OGB1 fluorescence measured over the entire field of view, from 5 different recordings. Continuous line: linear fit. Note the limited decrease of fluorescence over 80 minutes of recording. (**f**) Distribution of the diameter of the neuron bodies detected by the semi-automatic procedure.

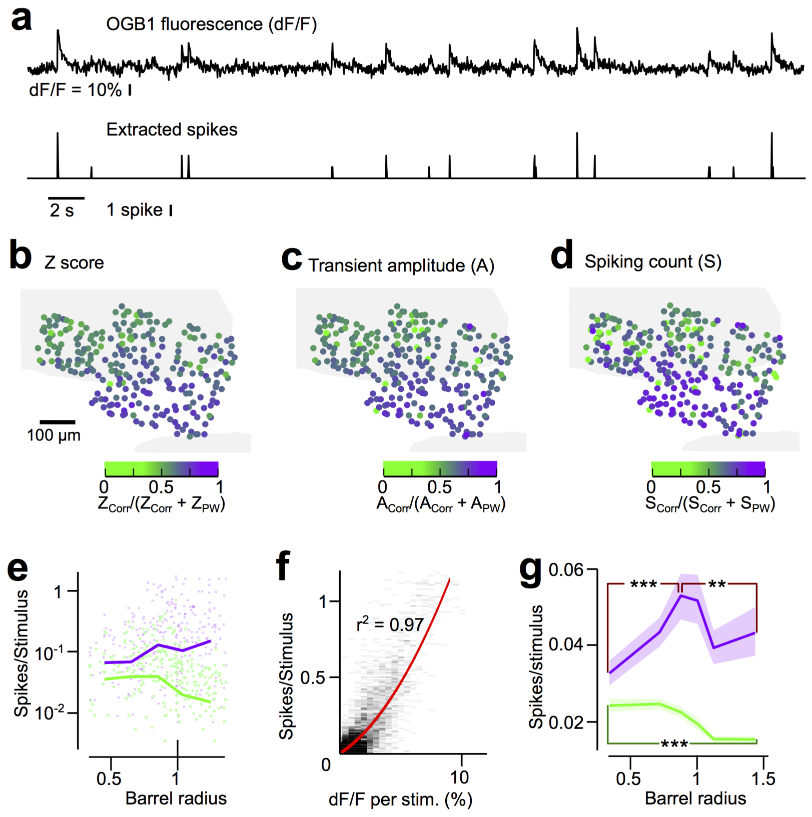

Supplementary Figure 4 Extraction of spiking activity from OGB-1 signal. (**a**) Representative example of OGB-1 fluorescence of a neuron (top) and the corresponding instantaneous firing rate extracted by the peeling algorithm (bottom). (**b**) Preference for correlated versus PW uncorrelated stimulations, measured on the Z score of 613 neurons recorded during one experiment, and localized on the barrel/septum anatomy. For this specific figure as well as the example in Fig. 1a, an independent analysis pipeline was used and manual instead of semi-automatic drawing of neurons borders was performed, allowing for the extraction of more neurons from this experiment. Note that only the semi-automatic analysis was used for all other analyses. (**c**) Same as **b**, measured on the calcium transients amplitude (mean dF/F over a 100 ms window post-stimulus, minus a baseline average dF/F measured over a 500ms window before stimulus). (**d**) Same as **b** for the average spike count increase observed over a 100ms window after stimulus onset compared to a 500 ms baseline. (**e**) For the same neurons as in **b-d**, radial distribution of spikes per stimuli for correlated (purple) and PW uncorrelated stimulations (green). Lines: local median spikes per stimuli. (**f**) Relationship between the mean spikes per stimuli extracted by the peeling algorithm and the mean fluorescence transient (dF/F) measured on 4 experiments. Red line: second order polynomial fit of the relationship. (**g**) Based on the fit in **f**, conversion of the population radial distribution of the mean fluorescent transients (shown in in **Fig. 2d**) into putative mean spikes per stimulus. Light background: bootstrap-derived 70% confidence interval on firing rate estimate. ***: Mann-Whitney p = 2.1E-6 for correlated and p = 5.3E-4 for uncorrelated-evoked. **: Mann-Whitney p = 2.0E-3.

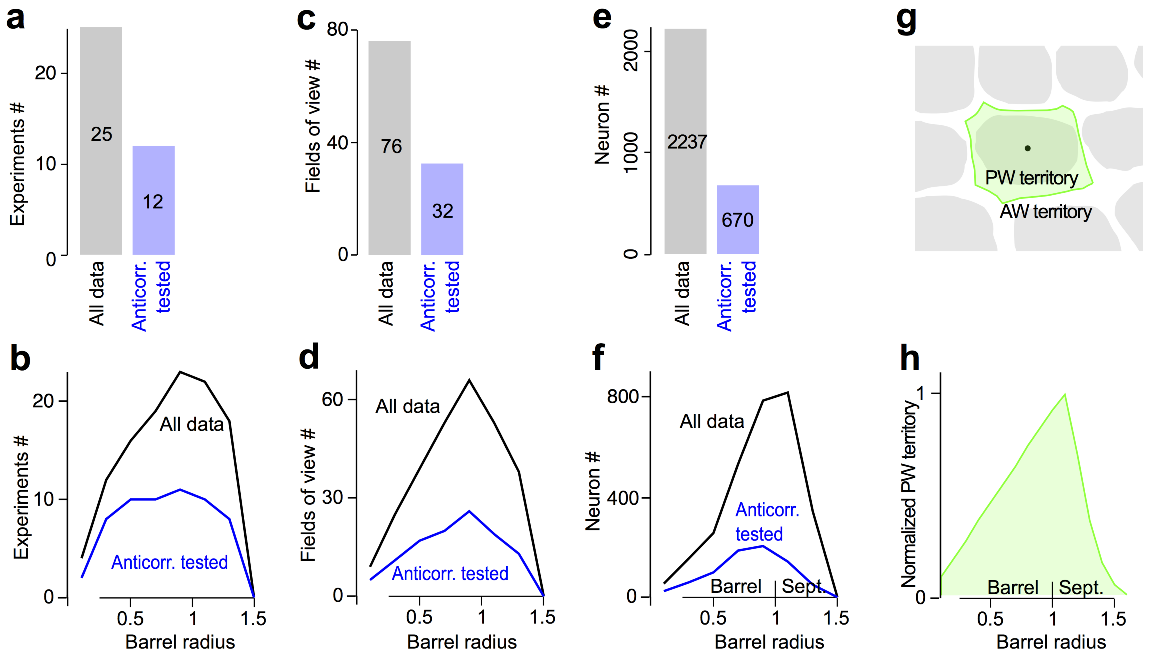

Supplementary Figure 5 Summary of the data, including the count of neurons that were sampled for each stimulus more than 30 times, and experiments/recordings that contain such neurons. Note the peak in sampling at the barrel/septum transition, due to barrel cortex geometry. To avoid this bias, instead of raw counts, proportions per bin are presented in the rest of the study. (**a**) Number of animals included in the project. Gray: all experiments. blue: experiments that included anti-correlated stimulations. (**b**) Number of experiments that have been recorded at a specific normalized barrel radius. (**c**) Same as **a** for fields of view (individual movies of neuronal activity). (**d**) Same as **b** for fields of view. (**e**) Same as **a** for neurons. (**f**) Same as **b** for neurons. (**g**) Example barrel field. Gray: barrels. White: septa. Green area: area that shares the same PW. (**h**) Average area that shares the same PW, measured across all central barrels of all experiments, and normalized by peak area. Note how the PW area radial distribution matches the radial distribution of neuron sampling in **f**. This suggests that the main reason for unequal radial sampling of neurons is the unequal radial distribution of the area that pertains to a given principal whisker territory.

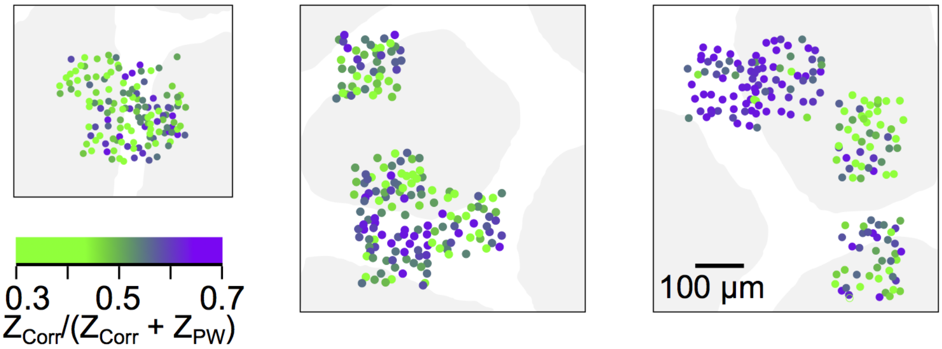

Supplementary Figure 6 Additional single experiment functional maps. Relative response to correlated versus uncorrelated PW stimulations of neurons recorded in three experiments in layer 2/3 and positioned with respect to the layer 4 barrel histological map. Gray: barrels. White: septa.

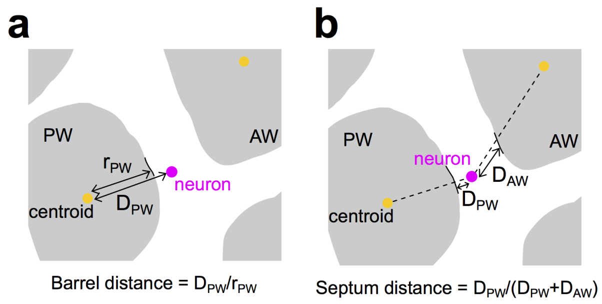

Supplementary Figure 7 Definition of the normalized coordinates. (**a**) The normalized barrel radius position of a neuron used through the paper is obtained by normalizing the distance from neuron to the centroid of the closest barrel (the PW barrel) by the distance from centroid to barrel/septum border on the same line. In this barrel radius space, barrel center corresponds to 0 and barrel edge to value 1. It can be extended into septa with values higher than 1. In this space, septum center is not defined. (**b**) The normalized septum position of a neuron as used in **Fig. 2h,i** is the distance between neuron and PW barrel border, normalized by the sum of the distances from neuron to principal whisker barrel border and neuron to adjacent whisker barrel border distance. In this space, 0 and 1 are the edges of the principal and adjacent whiskers, and 0.5 is the middle of the septum.
